# Patients' experiences with quality of hospital care: the Consumer Quality Index Cataract Questionnaire

**DOI:** 10.1186/1471-2415-7-14

**Published:** 2007-09-19

**Authors:** JH Stubbe, W Brouwer, DMJ Delnoij

**Affiliations:** 1 NIVEL (Netherlands Institute for Health Services Research), PO Box 1568, 3500 BN Utrecht, The Netherlands; 2 Department of Public Health, Erasmus MC, University Medical Center Rotterdam, The Netherlands; 3 Centre for Consumer Experience in Health Care, The Netherlands

## Abstract

**Background:**

Patients' feedback is of great importance in health care policy decisions. The Consumer Quality Index Cataract Questionnaire (CQI Cataract) was used to measure patients' experiences with quality of care after a cataract operation. This study aims to evaluate the reliability and the dimensional structure of this questionnaire and assesses its ability to measure differences between hospitals in patients' experiences with quality of care.

**Methods:**

Survey data of 4,635 respondents were available. An exploratory factor analysis was performed to evaluate the construct validity of the questionnaire and item-correlations and inter-factor correlations were calculated. Secondly, Cronbach's alpha coefficients were calculated to assess the internal consistency of the scales. Thirdly, to evaluate the ability of the questionnaire to discriminate between hospitals, multilevel analyses were performed with patients hierarchically nested within hospitals.

**Results:**

Exploratory factor analysis resulted in 14 quality of care items subdivided over three factors (i.e. communication with ophthalmologist, communication with nurses, and communication about medication). Cronbach's alpha coefficients of 0.89, 0.76 and 0.79 indicated good internal consistency. Multilevel analyses showed that the questionnaire was able to measure differences in patients' experiences with hospital care regarding communication with ophthalmologist and communication about medication. In addition, there was variation between hospitals regarding ophthalmologist ratings, hospital ratings and one dichotomous information item.

**Conclusion:**

These findings suggest that the CQI Cataract is a reliable and valid instrument. This instrument can be used to measure patients' experiences with three domains of hospital care after a cataract operation and is able to assess differences in evaluated care between hospitals.

## Background

Managed competition between health insurance organizations has been introduced in several industrialized countries [1]. In the Netherlands, consumers have a free choice of health insurers. Competition between insurers is possible with respect to premiums and quality of contracted providers. The basic benefits package is set by law [2] and is therefore no element in competition. Competition is expected to take place on the basis of prices (premiums), the service quality of insurers, and the quality of the care providers that they contract [3]. Therefore, transparent information about the performance of health care providers plays a key role in the Dutch system.

In Dutch hospitals, remuneration takes place on the basis of 'diagnosis treatment combinations' (DBCs). Prices for reimbursement are either based on fixed tariffs ('list A') or are subject of negotiations between health insurers and hospitals ('list B') [4]. Cataract surgery is a procedure on 'list B' for which individual consumers and insurance companies can "shop" among several providers. Comparative information about the performance and costs of these providers is therefore particularly valuable for individual consumers and for insurance companies.

To map the quality of care from a patient's perspective, specific instruments are needed. A wide variety of questionnaires is available, of which two 'families' of surveys stand out. One of these families of surveys is the Dutch QUOTE family with the acronym QUOTE standing for **QU**ality **O**f care **T**hrough the patients' **E**yes [5-11]. The QUOTE surveys distinguish themselves in two different ways. First of all, they not only measure experiences of health consumers, but also focus on the importance consumers attach to the different quality aspects of care. While experiences of patients will change when situations in health care services change, importance scores are less subject to situational changes as they are linked to the attitudes and opinions of patients [10]. Secondly, apart from the generic items that each questionnaire comprises, group-specific (e.g. focused on the elderly), care-specific (e.g. focused on physiotherapy), or disease-specific (e.g. focused on cataract patients) items are included in the questionnaire. The QUOTE Cataract [5,6] was developed to assess patient's experiences with quality of care after a cataract operation, however, one of the disadvantages of this questionnaire is that it uses answering categories that are internationally not widely used: no, not really, on the whole yes, and yes.

The second family of surveys is the Consumer Assessment of Healthcare Providers and Systems (CAHPS^®^) [12-18]. In contrast to the QUOTE methodology, these CAHPS questionnaires do not take the importance of quality aspects into account, and do not include group-, care- or disease-specific items. However, these questionnaires are widely used and translated into different languages [12,19-21]. Furthermore, the four-point Likert scale answering structure of the CAHPS questionnaires (never, sometimes, often, always), and the three-point scale (not a problem, a small problem, a big problem) affiliates well to international research.

To be able to measure both performance and importance of quality aspects on widely used scales, this paper proposes a new instrument for measuring patients' experiences with quality of care after a cataract operation: the Consumer Quality Index Cataract (CQI Cataract). The CQI Cataract is the first instrument that includes generic, and group-, care- or disease-specific items, measure the patients' experiences with and the importance patients attach to quality aspects, and uses the internationally accepted four-point Likert-scale answering structure. The development of the Consumer Quality Index Cataract Questionnaire (CQI Cataract) has been described in detail elsewhere [22].

The aim of this article is two-folded. First of all, we evaluate the psychometric properties of the CQI Cataract assessing patients' experiences with quality of care after a cataract surgery. The second aim of the paper is to assess the questionnaire's ability to measure differences in quality of care between hospitals. The two research questions derived from these two aims are: "*Is the CQI Cataract reliable and what is the dimensional structure of the instrument?*", and "*Does the CQI Cataract measure differences between hospitals in experiences with quality of care of patients who underwent cataract surgery?*".

## Methods

### Subjects

Case finding was done by contacting Dutch hospitals and through the administration of four Dutch insurance companies. Four Dutch health insurance companies recruited 5,323 patients for whom costs of cataract surgery were claimed within the last 12 months and 1,145 patients were directly recruited via hospitals after they had their cataract surgery, resulting in a total of 6,468 cataract patients who received a survey sent by mail. At the end of the data collection, 5,436 patients had returned the questionnaire (gross response rate = 84%). Of these patients, 447 respondents were not willing or able to participate. Patients were not included into the analyses if they responded negatively to the question whether or not they underwent a cataract operation (n = 32) or if this information was missing (n = 87). Furthermore, patients who stated that they did not answer the questions themselves (n = 203) or who filled in less than half of the core items (n = 32) were also excluded from the analyses. Therefore, a total of 801 subjects were excluded from the analyses, resulting in a sample of 4,635 patients (net response rate = 72%).

### Consumer Quality Index Cataract Questionnaire (CQI Cataract)

The development of the Dutch CQI Cataract was based on two different families of questionnaires [22]. Firstly, items from the QUOTE-Cataract [5,6] were used. The QUOTE-Cataract is a reliable, valid, and feasible instrument for assessing the quality of care from the perspective of cataract patients and is part of the QUOTE family of surveys [5-11,22,23]. These questionnaires conceptualize patients' experiences with quality of care in two dimensions: performance and importance [7]. Performance refers to the actual experience of patients with the quality aspects, and importance relates to the fact that people see some quality aspects as more significant than others. It reflects what people see as desired qualities in health care. The answering formats of importance items were: not important, fairly important, important, and extremely important. The original QUOTE answering formats for the performance items were: no, not really, on the whole yes, and yes. However, response options of the performance categories were adjusted to fit in with the internationally accepted four-point Likert-scale answering structure ranging from 'never' to 'always'. Some original QUOTE items could not be adjusted to fit the four-point scale, and were therefore transformed into a dichotomous variable (no/yes).

Besides the QUOTE-Cataract, the Dutch H-CAHPS measuring patients' experiences with quality of hospital care was used to generate items [19]. This questionnaire is part of the CAHPS family [12-14,16-18], and was shown to be a reliable, valid and feasible instrument for assessing the quality of hospital care from Dutch patients' perspectives [19]. Answering categories are based on a four-point Likert scale ranging from 'never' to 'always', or based on the three-point scale: 'not a problem', 'a small problem', and 'a big problem'. Items measuring quality of hospital care from the patient's perspective were selected.

Selecting items from the H-CAHPS and QUOTE-Cataract questionnaire resulted in the CQI Cataract, which consists of two questionnaires, i.e. the CQI Cataract Experience and the CQI Cataract Importance. The CQI Cataract Experience contains general items (e.g. age, education, ethnicity, and patient's health), three global ratings (of ophthalmologist, nurses and hospital), and 41 performance items referring to the actual experience of patients with the quality aspects (e.g. How often did the ophthalmologist treat you with respect). The global ratings range from 0 to 10, with a score of 10 indicating the best possible score.

The CQI Cataract Importance also comprises demographical items, and in addition consists of importance items asking how important cataract patients value the 41 quality aspects of the CQI Cataract Experience with answering categories ranging from not important to very important (e.g. My ophthalmologist treats me with respect). The outcome of the CQI Cataract is valuable, because it shows which quality aspects patients find important and how they evaluate these aspects.

### Analytic approach

In this paper we focus on patients' experiences with quality of hospital care and therefore, we only used the CQI Cataract Experience and selected the 41 items measuring quality aspects of hospital care. To evaluate the validity of this questionnaire, an exploratory factor analysis was conducted and item-total correlations correcting for item overlap were calculated. When variables are measured on a dichotomous (yes/no) scale, linear factor analysis (e.g. common factor analysis) may yield biased estimates of the factor structure [24,25]. Therefore, we did not include 21 dichotomous items measuring quality aspects.

The 20-item exploratory factor analysis was performed with a direct oblimin rotation. This oblique rotation was preferred to an orthogonal rotation (i.e. varimax), because it takes correlations between factors into account. An oblique rotation could also result in independent factors if that provides a better fit. The number of factors was determined by Kaiser's criterion [26]. In general, factor loadings are considered meaningful when they exceed 0.30 or 0.40 [27]. Therefore, items were only assigned to a factor if the magnitude of their factor loading exceeded 0.40. Item-total correlations (ITC) correcting for item overlap were calculated to evaluate the construct validity [28]. Correlations greater than 0.40 indicate good construct validity [29]. To get insight into the multidimensionality of the instrument, inter-factor correlations were computed. Correlations of less than 0.70 indicate that the constructed factors can be seen as separate scales [30].

Secondly, Cronbach's alpha coefficients of the different domains were calculated to evaluate the internal consistency of the questionnaire [31]. An alpha exceeding the value of 0.70 indicates that the scale is reliable [29]. After assigning the items to the different scales, mean sum scores were calculated by summing the responses to the items and dividing these sum scores by the number of items filled in. The higher the score on the domain, the more positive the patient's experience with quality of care.

Thirdly, to evaluate whether part of the variation in patients' evaluations of care is related to the hospital in which they were operated, multilevel analyses were performed.

Individual characteristics of patients were taken into account as case-mix adjusters to estimate the contribution of each characteristic. One of the goals of the questionnaire is to understand individual variations within hospitals. Therefore, it is important to investigate whether survey results might be influenced by factors that are not distributed randomly across hospitals, and if so, to adjust for differences in patient mix when making comparisons between hospitals. The case-mix adjusters consisted each of multiple answering categories and were recoded into dichotomous variables. The variable age consisted of eight answering categories ranging from "18–24 years" to "80 years and older", and was recoded into a variable consisting of two age groups "18–74 years" and "75 years and older". The variable education consisted of 11 categories ranging from no education to post academic education. The answering categories "no education" and "primary education" were recoded into the category "low education". All other educational levels were categorized as "high education". Self-reported health consisted of five answering categories, ranging from "excellent" to "poor". The three categories "excellent", "very good" and "good" were recoded into the category "good health" and "moderate" and "poor" were recoded into "bad health". We excluded respondents with missing values on age (N = 97), gender (N = 18), education (N = 183), and self-reported health status (N = 125).

Finally, only hospitals with a minimum of 10 patients in our dataset were included in the analyses, and therefore we had to exclude 176 respondents from 57 hospitals. In total, 599 respondents were excluded, resulting in 4,036 respondents from 57 different hospitals. The mean number of patients per hospital in our dataset was 45, with a minimum of 15 and a maximum of 141 patients. Three separate multilevel analyses were carried out on the following three domains of the CQI Cataract Experience: communication with ophthalmologist, communication with nurses, and communication about medication. Furthermore, we performed multilevel analyses on the three global ratings of ophthalmologist, nurses and hospital, because we hypothesised that these ratings may vary between hospitals.

The MLwiN software package was used [31], which deals with data that are hierarchically structured [32]. This means that the data are nested, i.e. ordered in such a way that one dependent variable is measured at the lowest level and exploratory variables at the same and higher levels. In our data, individual patients (level 1) are nested within hospitals (level 2). Our hypothesis is that experiences of patients measured at the first level depend partly on the hospital in which they were operated (second level). This should result in the fact that patients within the same hospital should agree more on experiences with quality of care than patients from different hospitals. The intra-class correlation (ICC) is an index of the ratio of the within-hospital variation and the between-hospital variation [33]. Values of the ICC range between 0 and 1. An ICC of zero indicates that the variance in patients' experiences of quality of care cannot be explained by the hospital in which they were operated.

The multilevel models used in the analyses can be viewed as hierarchical systems of regression coefficients. Regression coefficients and variance components are estimated based on the observed data. We fitted two different, nested models to the data. The first model is a random-intercept model in which no explanatory variables are included (Model 1). In this model, the variance of the dependent variable is partitioned into variance that can be attributed to the individual level, and to the hospital level.

Just like in regression analysis, explanatory variables can be used in the random-intercept model to try to explain part of the variability of the dependent variable [34]. These variables can be entered in the model as level-one (patients' characteristics) and level-two explanatory variables (hospital characteristics) and have the same interpretation as unstandardized regression coefficient in multiple regression models [34]. In the second model, individual characteristics (age, gender, education, and self-reported health), and one hospital characteristic (type of anaesthesia) were entered into the equation (Model 2).

Three different types of anaesthesia were used in the 57 Dutch hospitals, i.e. injection, topical preoperative drops and general anaesthesia. Currently, there is no consensus as to the optimal approach to anaesthesia [35]. However, since 2000, the use of topical preoperative drops has increased immensely [36], because topical anaesthesia bear no risk of injection-related complications and allow for a more rapid recovery after surgery [37], which may influence the experience of patients with the quality of care. Entering the percentage of preoperative drops used as anaesthesia in the hospital enables us to investigate whether patients are more positively about hospitals that use preoperative drops as anaesthetics compared to hospitals using other anaesthetics. Model 2 estimates how much of the variance is explained at the patient and hospital level after correcting for these individual and hospital variables and investigates whether survey results might be influenced by factors that are not distributed randomly across hospitals. Regression coefficients were estimated to get insight into the contribution of each characteristic.

Although we did not take the dichotomous variables into account in the factor analyses, they may be able to measure differences between hospitals in patients' experiences with quality of care. Of the dichotomous quality aspects, we selected one dichotomous item which was rated by patients as most important according to the CQI Cataract Importance and performed a logistic multilevel analysis. This information item asked patients if someone informed them about what to do in case of an emergency after the cataract operation. As with the previous multilevel analyses, first the random-intercept model was fitted to the data, followed by the model in which the individual and hospital characteristics were taken into account. Logistic multilevel analysis does not estimate regression coefficients, but calculates the odds ratios (OR). Furthermore, ρ is calculated, which can be interpreted as the ICC in linear multilevel analyses.

## Results

Individual characteristics of patients are displayed in Table 1. High ratings were given to ophthalmologist (mean = 8.8; standard deviation = 1.5), nurses (mean = 8.9; standard deviation = 1.2) and hospitals (mean 8.8; standard deviation = 1.3). These global ratings range from 0 to 10, with a score of 10 indicating the best possible score.

**Table 1:** Individual characteristics of the 4,036 respondents

Characteristics	Percentage
Age	
18–74	49.3
75+	50.7
	
Gender	
Male	37.8
Female	62.2
	
Education	
No or less than secondary education	33.6
Secondary or higher education	66.4
	
Self-reported health	
High	69.2
Low	30.8
	
Type of anaesthesia	
Injection anaesthesia	53.0
Preoperative drops	42.8
Narcosis	4.2

Data of 4,635 patients who underwent a cataract operation were used to perform an exploratory factor analysis. Four factors had eigenvalues greater than 1 (7.30, 1.71, 1.66, 1.07), and thus satisfied Kaiser's criterion. The amount of variance explained by the four factors was 58,8%. Table 2 gives an overview of the exploratory factor analysis.

**Table 2:** Factor loadings of quality aspects items according to the exploratory factor analysis with oblimin rotation

**nr**	**Item description**	**loading**	**α1**	**α2**	**ITC**
	*Factor 1: Communication with ophthalmologist*		0.89		
Q7	Ophthalmologist treated me with respect	0.88		0.88	0.66
Q8	Ophthalmologist listened carefully	0.90		0.87	0.73
Q9	Ophthalmologist explained things clearly	0.75		0.88	0.67
Q10	Ophthalmologist spent enough time	0.85		0.87	0.74
Q11	Ophthalmologist went seriously into merits of my questions	0.81		0.87	0.78
Q12	Ophthalmologist shared decision making	0.68		0.87	0.74
Q13	Ophthalmologist took specific wishes into account	0.68		0.87	0.74
Q16	Ophthalmologist talked about things that went wrong	0.40		0.91	0.49

	*Factor 2: Communication with nurses*		0.72		
Q30	Nurses treat me with respect	0.84		0.66	0.56
Q31	Nurses listened carefully	0.84		0.59	0.63
Q32	Nurses explained things clearly	0.80		0.62	0.56
Q38	Information was adequately coordinated*	0.41		0.76	0.41

	*Factor 3: Communication about medication*		0.79		
Q50	Questions asked about allergic iodine	0.91		0.69	0.65
Q51	Questions asked about allergic medication	0.92		0.58	0.74
Q52	Clear information about side-effects medication	0.61		0.83	0.51

	*Factor 4: Rest items*		0.52		
Q16	Ophthalmologist talked about things that went wrong	0.41		0.43	0.33
Q38	Information was adequately coordinated	0.53		0.36	0.44
Q39	Care was adequately coordinated	0.57		0.36	0.40
Q63	How often did insurers compensate for costs medication	0.46		0.61	0.12

Note: Cronbach's alpha of the whole scale (α1), Cronbach's alpha of the scale if item was deleted (α2), and corrected item total correlation (ITC) for the domains are shown. Factor loadings exceeding 0.40 are displayed. Q14, Q15, and Q49 were unrelated to any of the four factors and were not displayed.

* low corrected item total correlation (ITC) and an increase of the Cronbach's alpha of factor 2 if Q38 was deleted (α = 0.76) led to the exclusion of Q38 from factor 2.

Assigning items to a factor if the loading exceeded 0.40 resulted in the exclusion of Q14 ("How often did you enter the observation room of the ophthalmologist within 15 minutes of your appointment?"), Q15 ("How often did you have contact with the same ophthalmologist?"), and Q49 ("How often did nurses or other hospital employees clearly inform you about eye drops and/or eye crème that were prescribed?") from further scale construction. Furthermore, two items (Q16, and Q38) had loadings exceeding 0.40 on two different factors. Q16 loaded on factor 1 (0.40) and on factor 4 (0.41). Q38 loaded on factor 2 (0.41) and factor 4 (0.53).

Cronbach's alphas are displayed in the fourth column (α1) of Table 2 and their values range from 0.52 to 0.89. Factor 4 is the only scale that had a poor reliability coefficient (α = 0.52). Removing any of the items from this scale did not increase the coefficient to the threshold of 0.70 (see column 5 of Table 2). Therefore, it was decided to assign Q38 to factor 2 and Q16 to factor 1. However, low corrected item total correlation (ITC) and an increase of the Cronbach's alpha of factor 2 if Q38 was deleted (α = 0.79) led to the conclusion of excluding Q38 from factor 2.

The inter-factor correlation between communication with ophthalmologist and communication with nurses was 0.45. This correlation was 0.31 between communication with ophthalmologist and communication about medication and was 0.18 between communication with nurses and communication about medication. Correlations did not exceed the threshold of 0.70, which indicates that the scales could be read as separate scales.

Table 3 displays the results of the multilevel analyses for the three domains (i.e. communication with ophthalmologist, communication with nurses, and communication about medication) and for the three global ratings (of ophthalmologist, nurses and hospital). Model 1 and 2 showed that the variation between patients significantly differs from zero. Hospital variation was significantly different from zero for the two domains communication with ophthalmologist and communication about medication, and for the two global ratings of ophthalmologist and hospital. After including the individual characteristics, hospital variance decreased. However, hospitals still accounted for a significant part of the variance in patients' experiences with quality of care for four of the six outcome variables. Gender did not explain any variation between patients' experiences with quality of care. Except for a small negative effect on the domain communication about medication, percentage preoperative drops used in a hospital as anaesthetic did not have an effect on patients' experiences with quality of care. The regression coefficient for education was only significant on two of the six outcome variables. Subjective health and age explained part of the variance in patients' experiences for respectively five and four outcome variables. ICC's for models 2 ranged from 0.02 to 0.03.

**Table 3:** Model fitting results of the multilevel analyses for the domains communication with ophthalmologist, communication with nurses, and communication about medication and for the global rating of ophthalmologist, nurses and hospitals (standard errors added in parentheses)

**Outcome variable**	**intercept**	**β age**^1^	**β gender**^1^	**β education**^1^	**β health**^1^	**β% drops**	**Var patients**	**Var hospital**	**ICC**^2^
*Domain ophthalmologist (N = 3942)*									
Model 1	3.640 (0.014)*	-	-	-	-	-	0.246 (0.006)*	0.006 (0.002)*	0.02
Model 2	3.687 (0.031)*	-0.005 (0.016)	0.017 (0.016)	-0.031 (0.017)	-0.102 (0.017)*	0.000 (0.000)	0.243 (0.006)*	0.007 (0.002)*	0.03
									
*Domain nurses (N = 3925)*									
Model 1	3.726 (0.009)*	-	-	-	-	-	0.196 (0.004)*	0.002 (0.001)	n.c.
Model 2	3.767 (0.024)*	-0.020 (0.014)	0.005 (0.015)	-0.016 (0.015)	-0.089 (0.015)*	0.000 (0.000)	0.194 (0.004)*	0.002 (0.001)	n.c
									
*Domain medication (N = 3887)*									
Model 1	2.670 (0.033)*	-	-	-	-	-	1.246 (0.028)*	0.038 (0.011)*	0.03
Model 2	3.015 (0.070)*	-0.214 (0.036)*	-0.035 (0.037)	-0.166 (0.039)*	-0.020 (0.039)	-0.002 (0.001)*	1.232 (0.028)*	0.031 (0.010)*	0.02
									
*Rating ophthalmologist (N = 3940)*									
Model 1	8.791 (0.039)*	-	-	-	-	-	2.124 (0.048)*	0.046 (0.015)*	0.02
Model 2	8.894 (0.088)*	0.104 (0.047)*	0.013 (0.048)	-0.106 (0.051)	-0.308 (0.051)*	0.000 (0.001)	2.101 (0.048)*	0.045 (0.015)*	0.02
									
*Rating nurses (N = 3910)*									
Model 1	8.916 (0.027)*	-	-	-	-	-	1.466 (0.033)*	0.016 (0.007)*	0.01
Model 2	8.943 (0.065)*	0.084 (0.039)*	0.004 (0.040)	-0.086 (0.042)	-0.219 (0.043)*	0.001 (0.001)	1.455 (0.033)*	0.011 (0.006)	n.c
									
*Rating hospital (N = 3970)*									
Model 1	8.775 (0.036)*	-	-	-	-	-	1.730 (0.039)*	0.041 (0.013)*	0.02
Model 2	8.987 (0.080)*	0.064 (0.042)*	-0.040 (0.043)	-0.127 (0.045)*	-0.306 (0.046)*	-0.001 (0.001)	1.709 (0.039)*	0.037 (0.013)*	0.02

*p < 0.05

^1^Reference group age = younger than 75; reference group gender = males; reference group education = low education; reference group health = good health

^2^ICC (intra-class correlation) = Var hospital/(Var patients + Var hospital)

n.c. = not calculated. The variance explained by the hospital level is not significant and, therefore, the ICC is not calculated.

The results of the logistic multilevel analysis on the dichotomous information item are shown in Table 4. The probability of receiving emergency information was high (i.e. π = 0.79 for model 1). Furthermore, μ_0j _showed that there was variation between hospitals in patients' experiences with care. Two individual variables significantly explained part of the variation between patients' evaluations. The OR for age and subjective health were 0.64 and 0.61, respectively.

**Table 4:** Model fitting results of the logistic multilevel analyses for the dichotomous variable "Did someone inform you on what to do in case of an emergency after the cataract operation?"

**Outcome variable (N = 3,871)**	**Model 1**	**Model 2**
Probability (π)^1^	0.79 (0.76, 0.81)	0.84 (0.80, 0.88)
OR age (18–74 = reference)^1^	-	0.64 (0.54, 0.75)*
OR sex (males = reference)^1^	-	1.09 (0.92, 1.28)
OR education (low education = reference)^1^	-	0.91 (0.76, 1.08)
OR health (high = reference)^1^	-	0.61 (0.72, 0.85)*
OR anaesthesia (injection = reference)^1^		0.98 (0.81, 1.19)
Variance hospitals^2^	0.22 (0.07)*	0.23 (0.07)*
Variance patients^2^	0.98 (0.02)^3^	0.98 (0.02)^3^
ρ^4^	0.06	0.07

*p < 0.05

^1^OR = Odds ratio and 95% confidence intervals added in parentheses

^2^Standard errors added in parentheses

^3^By definition, individual level variance is one if the binomial distribution holds

^4^ρ can be interpreted as the ICC in linear multilevel analyses

## Discussion

In this paper, we investigated the construct validity and internal consistency of the CQI Cataract and evaluated its ability to discriminate between hospitals in quality of care. Exploratory factor analysis showed that 14 items could be subdivided into the following three scales: communication with ophthalmologist, communication with nurses, and communication about medication. Cronbach's alpha coefficients ranged from 0.76 to 0.89, indicating good internal consistency. Item-total correlations corrected for overlap all exceeded the threshold of 0.40, suggesting good construct validity. Multidimensionality of the scales was supported by the inter-factor correlations, which were all smaller than the commonly used rule of thumb of 0.70. However, these correlations were not equal to zero and therefore, the use of an oblique rotation (i.e. oblimin rotation) was justified. Three items did not meet the psychometric standards (Q14, Q15, and Q49). However, these items might give important information on the quality of care. Therefore, we are reluctant to eliminate these items from the questionnaire, even though they cannot be assigned to a certain domain.

Multilevel analyses showed that hospitals accounted for part of the variance in patients' experiences with quality of care on the following five outcome variables: communication with ophthalmologist, communication about medication, global rating of ophthalmologist, and global hospital rating, and the dichotomous variable about emergency information. Experiences of patients with communication with nurses and global ratings of nurses did not differ significantly between hospitals. This is not in agreement with result of a study on patients' experiences with quality of care after a total hip or knee arthroplasty, which showed that communication with nurses differed significantly between hospitals [38]. This discrepancy might be explained by the fact that a total hip or knee arthroplasty needs more hospital follow-up than a cataract operation, and therefore, nurses play a less important role in the recovery process of cataract patients.

Multilevel analyses showed that self-reported health and age were significantly associated with patients' health care evaluations on most of the outcome variables. Patients with low levels of self-reported health gave lower ratings and scored lower on the quality domains, except for the domain communication about medication. A review by Pascoe and colleagues [39] showed similar results and found that patients with better health tend to be more satisfied with evaluations of health centres. Gender did not explain any variation in patients' experiences with quality of hospital care.

Furthermore, we found that the individual characteristic age gave mixed results. Older patients scored higher on global ratings of care and lower on communication about medication. Contrary to our results, several studies showed that age was the variable having the most consistent effect, being associated with higher satisfaction with care [19,39]. The reason for not being able to replicate this finding in our sample might be the fact that our respondents were part of a homogenous age group. More than 50% of the cataract patients were older than 74 years and less than 5% was younger than 55 years. In both studies that found an age effect, age of the sample was more heterogeneously distributed than in our sample.

Cataract surgery is performed using general or local anaesthetics. Local anaesthetic may be administered through either injection (peribular, retrobullar, subconjunctival, etc.) or the use of topical anaesthetic drops (with or without intracameral application) [35]. Retrobulbar and peribulbar blocks provide better pain control during surgery than topical anaesthesia. However, the application of anaesthesia by injection is more painful than by topical means for patients not receiving sedation [35]. Furthermore, topical anaesthesia does not cause injection-related complications and there is a more rapid recovery after surgery [37]. Despite these differences, there was no variation in patients' experiences with quality of care between hospitals using preoperative drops on a small scale compared to hospitals that used preoperative drops to a large degree. Although not described in the results section, instead of using type of anaesthesia as a hospital characteristic, we also entered the type of anaesthesia as a patient's characteristic in the model. Topical anaesthesia did not explain any variation between patients and therefore, it seemed not to influence patients' experiences with care. This might be explained by the fact that due to small numbers we had to combine injection and general anaesthetics into one group and we were not able to make distinctions between the different types of injection (peribular, retrobullar, subconjunctival) and topical anaesthetic drops (with or without intracameral application).

For the multilevel analysis an arbitrary cut off point of 10 patients per hospital was used, resulting in a dataset that consisted of 57 hospitals. Several papers investigated the minimal group size and the minimal number of groups needed in multilevel analysis. Kreft and De Leeuw [40] pointed out that the size of the highest and lowest number of groups is based on literature: 30 groups are mentioned as minimum, while 100 groups are seen as sufficient. In practice, 50 groups is a frequently occurring number. The latter is in agreement with our number of 57 hospitals. Similarly, the size of the group is also chosen on the basis of the literature. A group size of 30 is normal in educational research, and a group size of five is normal in family research and in longitudinal research. Our arbitrary cut-off point of 10 patients per hospital fits well in this range.

In general, Dutch cataract patients are very satisfied with the quality of care given by hospitals. As a result the mean global ratings for hospitals, nurses and ophthalmologist by cataract patients are high. A study by Stubbe and colleagues showed that Dutch patients who underwent a total hip or knee operation also evaluated the quality of care very positively [38]. As in our sample, respondents were part of a homogenous sample of older patients, which might explain the high ratings of quality of care. Two studies confirm this hypothesis [41,42]. Both studies found that older patients are more satisfied and suggest that this can be explained by the fact that older people are generally mellow and accepting, feel more reluctant than younger patients to pass negative judgement on their care, have lower expectations and therefore are more satisfied. Two other studies provided another reason for the higher satisfaction ratings of older patients. [43,44]. These two studies showed that older patients happened to be treated in a more thorough or responsive manner than younger patients.

There is little variation between patients in experiences with quality of care and therefore variation in evaluations of care is small between hospitals, which is reflected in the small intra-class correlation (ICC) ranging from 0.02 to 0.03. However, mostly ICCs are lower, for example, the median ICC calculated for more than 1000 primary care variables was 0.01 [45].

Similar multilevel results were found for the dichotomous information variable. Age and self-rated health explained part of the probability of receiving information about what patients should do in case of an emergency after their cataract operation. Younger and healthier patients were more likely to report having received information than older and unhealthier patients. However, after entering these variables in the model still part of the probability of receiving information was explained by the hospital in which patients were operated. Therefore, we can conclude that on an item level the CQI Cataract is able to measure differences between hospitals in patients' evaluation of quality of care.

## Conclusion

The CQI Cataract questionnaire is a reliable and valid instrument for measuring patients' experience with quality of care after a cataract operation. Use of this instrument allows comparisons between hospitals on two domains (i.e. communication with ophthalmologist, and communication about medication), two global ratings (i.e. of hospital and ophthalmologist rating), and on at least one item.

## Competing interests

The author(s) declare that they have no competing interests.

## Authors' contributions

JS performed the psychometric and multilevel analyses and was the principal author of the paper. WB constructed the questionnaire, collected the data and performed the analyses regarding construct validity and internal consistency. DD designed the study, supervised the project and assisted in writing the final draft of the paper. All authors read and approved the final manuscript.

## Pre-publication history

The pre-publication history for this paper can be accessed here:


